# Supplementary material for: ZBTB16/PLZF regulates juvenile spermatogonial stem cell development through an extensive transcription factor poising network
Source: Nat Struct Mol Biol. 2025 Mar 3;32(7):1213–26. doi: 10.1038/s41594-025-01509-5 (PMC12263333; doi:10.1038/s41594-025-01509-5)
Supplement: Supplementary file 1 — Reporting Summary [file 41594_2025_1509_MOESM1_ESM.pdf]

nature portfolio

Corresponding author(s): Bradley R. Cairns

Last updated by author(s): Dec 30, 2024

Reporting Summary

Nature Portfolio wishes to improve the reproducibility of the work that we publish. This form provides structure for consistency and transparency in reporting. For further information on Nature Portfolio policies, see our Editorial Policies and the Editorial Policy Checklist.

Statistics

For all statistical analyses, confirm that the following items are present in the figure legend, table legend, main text, or Methods section.

n/a	Confirmed	
☐	☒	The exact sample size (*n*) for each experimental group/condition, given as a discrete number and unit of measurement
☐	☒	A statement on whether measurements were taken from distinct samples or whether the same sample was measured repeatedly
☐	☒	The statistical test(s) used AND whether they are one- or two-sided<br>*Only common tests should be described solely by name; describe more complex techniques in the Methods section.*
☒	☐	A description of all covariates tested
☐	☒	A description of any assumptions or corrections, such as tests of normality and adjustment for multiple comparisons
☐	☒	A full description of the statistical parameters including central tendency (e.g. means) or other basic estimates (e.g. regression coefficient) AND variation (e.g. standard deviation) or associated estimates of uncertainty (e.g. confidence intervals)
☐	☒	For null hypothesis testing, the test statistic (e.g. F, t, r) with confidence intervals, effect sizes, degrees of freedom and P value noted<br>*Give P values as exact values whenever suitable.*
☒	☐	For Bayesian analysis, information on the choice of priors and Markov chain Monte Carlo settings
☒	☐	For hierarchical and complex designs, identification of the appropriate level for tests and full reporting of outcomes
☒	☐	Estimates of effect sizes (e.g. Cohen's *d*, Pearson's *r*), indicating how they were calculated

Our web collection on statistics for biologists contains articles on many of the points above.

Software and code

Policy information about availability of computer code

Data collection	RNA-seq and ChIP-seq reads were aligned to the mm10 mouse genome using Novoalign (v4.04.01) with default parameters. Differential gene expression analysis was performed using USeq (v8.9.6), incorporating DESeq2 (v1.42.1). ChIP-seq peaks were identified with USeq (v8.9.6) and MACS2 (v2.1.1) using default settings. Genome browser visualizations were generated with IGV (v2.16.2).<br>Published data were retrieved from the NCBI Gene Expression Omnibus (GEO) Sequence Read Archive (SRA) using GNU Wget and SRA Toolkit (v2.10.8), with the fasterq-dump utility for sequence extraction.<br>Hi-C data were processed using Juicer (v1.5) with BWA (v0.7.3a) for alignment. The resulting .hic file was generated using Cooler (v0.8.11) and visualized with Cooler tools (coolup.py, v0.9.5).
Data analysis	http://novocraft.com (Novoalign v4.04.01)<br>https://www.gnu.org/software/wget/ (GNU Wget)<br>https://hpc.nih.gov/apps/sratoolkit.html (SRA Toolkit v2.10.8)<br>https://useq.sourceforge.net (USeq v8.9.6)<br>https://hbctraining.github.io/Intro-to-ChIPseq/lessons/05_peak_calling_macs.html (MACS2 v2.1.1)<br>http://www.htslib.org (Samtools v1.19)<br>https://bioconductor.org/packages/release/bioc/html/DESeq2.html (DESeq2 v1.42.1)<br>https://broadinstitute.github.io/picard/ (Picard v2.26.3)<br>https://deeptools.readthedocs.io/en/latest/ (deepTools v3.5.4)<br>https://igv.org (IGV v2.16.2)<br>https://bedtools.readthedocs.io/en/latest/ (BEDTools v2.29.0)<br>https://pantherdb.org (PANTHER v17.0)<br>https://bioinforx.com/apps/venn_overlap.php(BxToolBox: Venn diagram)

https://github.com/tjparnell/biotoolbox (biotoolbox v1.69)
https://meme-suite.org/meme/tools/meme-chip (MEME-ChIP v11)
https://groups.csail.mit.edu/cgs/gem/ (GEM v2.6)
https://ggplot2.tidyverse.org (ggplot2 v3.5.1)
https://cran.r-project.org/web/packages/pheatmap/index.html (pheatmap v1.0.12)
https://www.bdbiosciences.com/en-us/products/software/instrument-software/bd-facsdiva-software (FACSDiva v8.01)
https://www.flowjo.com (FlowJo v9.9)
https://imagej.net/software/fiji/ (Image J-Fiji v1.53t)
http://nemates.org/MA/progs/overlap_stats.html (hypergeometric probability tests)
https://github.com/aidenlab/juicer/releases (juicer v1.5)
https://pypi.org/project/cooler/ (cooler v0.8.11)
https://pypi.org/project/coolpuppy/ (coolup.py v0.9.5)

For manuscripts utilizing custom algorithms or software that are central to the research but not yet described in published literature, software must be made available to editors and reviewers. We strongly encourage code deposition in a community repository (e.g. GitHub). See the Nature Portfolio guidelines for submitting code & software for further information.

Data

Policy information about availability of data

All manuscripts must include a data availability statement. This statement should provide the following information, where applicable:

- Accession codes, unique identifiers, or web links for publicly available datasets
- A description of any restrictions on data availability
- For clinical datasets or third party data, please ensure that the statement adheres to our policy

The datasets generated and/or analyzed during this study are publicly available on the GEO database under the following accession numbers: bulk RNA-seq and ChIP-seq data (GSE202819) and Hi-C data (GSE244681). All data sources, including the mm10 genome assembly, are appropriately cited and referenced in the manuscript.

Research involving human participants, their data, or biological material

Policy information about studies with human participants or human data. See also policy information about sex, gender (identity/presentation), and sexual orientation and race, ethnicity and racism.

Reporting on sex and gender	N/A
Reporting on race, ethnicity, or other socially relevant groupings	N/A
Population characteristics	N/A
Recruitment	N/A
Ethics oversight	N/A

Note that full information on the approval of the study protocol must also be provided in the manuscript.

Field-specific reporting

Please select the one below that is the best fit for your research. If you are not sure, read the appropriate sections before making your selection.

☒ Life sciences ☐ Behavioural & social sciences ☐ Ecological, evolutionary & environmental sciences

For a reference copy of the document with all sections, see nature.com/documents/nr-reporting-summary-flat.pdf

Life sciences study design

All studies must disclose on these points even when the disclosure is negative.

Sample size	Sample sizes were determined based on standard practices in the field and the feasibility of the experimental design. For each experiment, we ensured that the number of biological replicates was sufficient to capture variability and allow robust statistical analyses. Although no formal sample size calculation was performed, the chosen sample sizes are consistent with similar studies and provide adequate statistical power to support the conclusions. The sufficiency of these sample sizes was confirmed through statistical tests that showed clear differentiation between experimental groups. For all genomics experiments, ChIP-seq and Hi-C were performed in duplicate, while bulk RNA-seq experiments were performed in triplicate to ensure reproducibility. Flow cytometry experiments were conducted in three biological replicates. Microscopy experiments were conducted in three biological replicates, with quantifications based on at least 300 seminiferous tubules per measurement to ensure accurate and reliable assessment of trends.
Data exclusions	No data were excluded from analyses.

Replication	We verified consistent results across three independent biological replicates for flow cytometry and immunofluorescence analyses. Additionally, the ChIP-seq experiments for ZBTB16, SALL4, and SOX3 were conducted with two independent biological replicates, confirming consistency through Spearman correlation analysis using plotCorrelation (deepTools) and pairwise R square values among the three independent biological replicates of RNA-seq.
Randomization	Randomization was not applicable to this study as the experimental groups (e.g., genotypes or developmental stages) were predefined by the study design and biological context. Covariates were controlled by using consistent experimental conditions, including identical protocols for sample preparation, data collection, and analysis across all groups. Biological replicates were derived independently to account for natural variability, ensuring the robustness of the findings.
Blinding	Blinding was not performed in this study because the experimental groups (e.g., genotypes or developmental stages) were explicitly predefined and inherently distinguishable, such as through genetic markers or developmental time points. The nature of the study required precise identification of these groups during sample processing and analysis. To minimize bias, consistent protocols were applied across all groups, and data analysis was conducted using automated and objective computational tools wherever possible.

Reporting for specific materials, systems and methods

We require information from authors about some types of materials, experimental systems and methods used in many studies. Here, indicate whether each material, system or method listed is relevant to your study. If you are not sure if a list item applies to your research, read the appropriate section before selecting a response.

Materials & experimental systems

n/a	Involved in the study
☐	☒ Antibodies
☒	☐ Eukaryotic cell lines
☒	☐ Palaeontology and archaeology
☐	☒ Animals and other organisms
☒	☐ Clinical data
☒	☐ Dual use research of concern
☒	☐ Plants

Methods

n/a	Involved in the study
☐	☒ ChIP-seq
☐	☒ Flow cytometry
☒	☐ MRI-based neuroimaging

Antibodies

Antibodies used

We provide a comprehensive list of all antibodies used in this study in Extended Data Table 1, including details on the manufacturer, catalog number, and specific applications.

Validation

All antibodies employed in this study are commercially available and were validated by the manufacturer. Validation of rabbit anti-ZBTB16, goat anti-ZBTB16 and mouse anti-SOX3 antibodies included the use of relevant mutant animals in this study. In details:

Host organism Target Species Manufacturer Catalog number RRID Validation statement
mouse ACTB mouse Proteintech Cat# 66009-1-lg AB_2782959 https://www.ptglab.com/products/Pan-Actin-Antibody-66009-1-Ig.htm
rabbit ADAMTS5 mouse Thermo Fisher Scientific Cat# PA5-27165 AB_2544641 https://www.thermofisher.com/antibody/product/ADAMTS5-Antibody-Polyclonal/PA5-27165
mouse CCND1 mouse Santa Cruz Biotechnology Cat# sc-8396 AB_627344 https://www.scbt.com/p/cyclin-d1-antibody-a-12?gad_source=1&gclid=Cj0KCQjwqP2pBhDMARIsAJQ0CzrS5XHH5sEjTc8G84h58NdC2Ec1dlLl89WhA0bEHpE9c9fwdiHLTboaAp5UEALw_wcB
rat CCND2 mouse Santa Cruz Biotechnology Cat# sc-452 AB_627350 https://www.scbt.com/p/cyclin-d2-antibody-34b1-3
goat GATA4 mouse Santa Cruz Biotechnology Cat# sc-1237 AB_2108747 https://www.scbt.com/p/gata-4-antibody-c-20
rabbit H3K27me3 mouse EMD Millipore Cat# 07-449 AB_310624 https://www.emdmillipore.com/US/en/product/Anti-trimethyl-Histone-H3-Lys27-Antibody,MM_NF-07-449?ReferrerURL=https%3A%2F%2Fwww.google.com%2F
rabbit H3K4me3 mouse Active Motif Cat# 39159 AB_2615077 https://www.activemotif.com/catalog/details/39159/histone-h3-trimethyl-lys4-antibody-pab
rabbit H3K9me3 mouse Active Motif Cat# 39161 AB_2532132 https://www.activemotif.com/catalog/details/39161/histone-h3-trimethyl-lys9-antibody-pab
rat KIT/CD117 mouse Miltenyi Biotec Cat# 130-091-224 AB_2753213 https://www.miltenyibiotec.com/US-en/products/cd117-microbeads-mouse.html#130-091-224
rat KIT/CD117-PE mouse Thermo Fisher Scientific Cat# 12-1171-81 AB_465812 https://www.thermofisher.com/antibody/product/CD117-c-Kit-Antibody-clone-2B8-Monoclonal/12-1171-81
goat LIN28A mouse R&D systems Cat# AF3757 AB_2234537 https://www.rndsystems.com/products/human-lin-28a-antibody_af3757
rabbit Phospho-CDK4 (Thr172) mouse Thermo Fisher Scientific Cat# 702556 AB_2632989 https://www.thermofisher.com/antibody/product/Phospho-CDK4-Thr172-Antibody-clone-9H2L7-Recombinant-Monoclonal/702556
rabbit Phospho-CDK6 mouse Thermo Fisher Scientific Cat# PA537517 AB_2554126 https://www.thermofisher.com/antibody/product/Phospho-CDK6-Tyr13-Antibody-Polyclonal/PA5-37517
mouse Phospho-H2AX mouse Abcam Cat# ab26350 AB_470861 https://www.abcam.com/products/primary-antibodies/gamma-h2ax-phospho-s139-antibody-9f3-ab26350.html
rabbit Phospho-H2AX (S136, 20E3) mouse Cell Signaling Cat# 9718 AB_2118009 https://www.cellsignal.com/products/primary-

antibodies/phospho-histone-h2a-x-ser139-20e3-rabbit-mab/9718
mouse Pan-RNAPol2 mouse "Active Motif" Cat# 39097 AB_2732926 https://www.activemotif.com/catalog/details/39097/rna- pol-ii-antibody-mab
rabbit RNAPol2 CTD phospho Ser5 mouse "Active Motif" Cat# 39233 AB_2793198 https://www.activemotif.com/catalog/details/39233/rna- pol-ii-ctd-phospho-ser5-antibody-pab
rat RNAPol2 CTD phospho Ser2 mouse "Active Motif" Cat# 61083 AB_2687450 https://www.activemotif.com/catalog/details/61083/rna- pol-ii-ctd-phospho-ser2-antibody-mab
rabbit SALL4 mouse Abcam Cat# ab29112 AB_777810 https://www.abcam.com/products/primary-antibodies/sall4-antibody-ab29112.html
rabbit SOX3 mouse EMD Millipore Cat# AB5772 AB_2302597 https://www.sigmaaldrich.co.th/th_en/ab5772-th
mouse SOX3 mouse Santa Cruz Biotechnology Cat# sc-101155 AB_2195961 https://www.scbt.com/p/sox-3-antibody-16-c2
mouse SYCP3 mouse Abcam Cat# ab97672 AB_10678841 https://www.abcam.com/products/primary-antibodies/scp3-antibody-cor-10g117-ab97672.html
mouse SYCP3 mouse Santa Cruz Biotechnology Cat# sc-74569 AB_2197353 https://www.scbt.com/p/scp-3-antibody-d-1
mouse TBP mouse EMD Millipore Cat# MAB3658 AB_2200056 https://www.emdmillipore.com/US/en/product/Anti-TATA-Binding-Protein-Antibody,MM_NF-MAB3658
rat THY1/CD90.2 mouse Miltenyi Biotec Cat# 130-049-101 AB_3073748 https://www.miltenyibiotec.com/US-en/products/cd90-2-microbeads-mouse.html
rat THY1/CD90.2-PE-Cy7 (53-2-1) mouse Thermo Fisher Scientific Cat# 25-0902-81 AB_469641 https://www.thermofisher.com/antibody/product/CD90-2-Thy-1-2-Antibody-clone-53-2-1-Monoclonal/25-0902-81
mouse UTF1 mouse EMD Millipore Cat# MAB4337 AB_827541 https://www.emdmillipore.com/US/en/product/Anti-UTF-1-Antibody-clone-5G10.2,MM_NF-MAB4337
rabbit VERSICAN V0, V1 Neo mouse Thermo Fisher Scientific Cat# PA1-1748A AB_2304324 https://www.thermofisher.com/antibody/product/PA1-1748A.html?ef_id=Cj0KCQjwqP2pBhDMARIsAJQ0CzqyYcJEbrSNQY5iQofd3Z0IO3xyge1G_agd3S9qnkk-xTCRdJBGndwaAhF_EALw_wcB:G:s&s_kwcid=AL!3652!3!459737518508!!!g!!!10950825775!106531320406&cid=bid_pca_aup_r01_co_cp1359_pjt0000_bid00000_0se_gaw_dy_pur_con&gad_source=1&gclid=Cj0KCQjwqP2pBhDMARIsAJQ0CzqyYcJEbrSNQY5iQofd3Z0IO3xyge1G_agd3S9qnkk-xTCRdJBGndwaAhF_EALw_wcB
goat ZBTB16 mouse Santa Cruz Biotechnology Cat# sc-11146 AB_2218938 https://www.scbt.com/p/plzf-antibody-n-21
rabbit ZBTB16 mouse Santa Cruz Biotechnology Cat# sc-22839 AB_2304760 https://www.scbt.com/p/plzf-antibody-h-300
goat Rabbit IgG-HRP mouse Bio-Rad Cat# 170-6518 AB_11125338 https://www.bio-rad.com/en-us/sku/1706518-goat-anti-rabbit-igg-ap-conjugate?ID=1706518
bovine Goat IgG-HRP mouse Santa Cruz Biotechnology Cat# sc-2354 https://www.scbt.com/p/mouse-anti-goat-igg-hrp?srsltid=AfmBOoqyWfLHmZRs1kYZJXmqq1_4Dmit850qpbpwc0aDZljGZFxrPo7m
donkey Alex Fluor 488 donkey anti-mouse IgG mouse Thermo Fisher Scientific Cat# A21202 AB_141607 https://www.thermofisher.com/antibody/product/Donkey-anti-Mouse-IgG-H-L-Highly-Cross-Adsorbed-Secondary-Antibody-Polyclonal/A-21202
donkey Alex Fluor 594 donkey anti-mouse IgG mouse Thermo Fisher Scientific Cat# A21203 AB_2535789 https://www.thermofisher.com/antibody/product/Donkey-anti-Mouse-IgG-H-L-Highly-Cross-Adsorbed-Secondary-Antibody-Polyclonal/A-21203
donkey Alex Fluor 647 donkey anti-mouse IgG mouse Thermo Fisher Scientific Cat# A31571 AB_162542 https://www.thermofisher.com/antibody/product/Donkey-anti-Mouse-IgG-H-L-Highly-Cross-Adsorbed-Secondary-Antibody-Polyclonal/A31571
donkey Alex Fluor 488 donkey anti-rabbit IgG mouse Thermo Fisher Scientific Cat# A21206 AB_2535792 https://www.thermofisher.com/antibody/product/Donkey-anti-Rabbit-IgG-H-L-Highly-Cross-Adsorbed-Secondary-Antibody-Polyclonal/A-21206
donkey Alex Fluor 594donkey anti-rabbit IgG mouse Thermo Fisher Scientific Cat# A21207 AB_141637 https://www.thermofisher.com/antibody/product/Donkey-anti-Rabbit-IgG-H-L-Highly-Cross-Adsorbed-Secondary-Antibody-Polyclonal/A-21207
donkey Alex Fluor 647 donkey anti-rabbit IgG mouse Thermo Fisher Scientific Cat# A31573 AB_2536183 https://www.thermofisher.com/antibody/product/Donkey-anti-Rabbit-IgG-H-L-Highly-Cross-Adsorbed-Secondary-Antibody-Polyclonal/A-31573
donkey Alex Fluor 594 donkey anti-goat IgG mouse Thermo Fisher Scientific Cat# A11058 AB_2534105 https://www.thermofisher.com/antibody/product/Donkey-anti-Goat-IgG-H-L-Cross-Adsorbed-Secondary-Antibody-Polyclonal/A-11058
donkey Alex Fluor 488 donkey anti-rat IgG mouse Thermo Fisher Scientific Cat# A21208 AB_2535794 https://www.thermofisher.com/antibody/product/Donkey-anti-Rat-IgG-H-L-Highly-Cross-Adsorbed-Secondary-Antibody-Polyclonal/A-21208

Animals and other research organisms

Policy information about studies involving animals; ARRIVE guidelines recommended for reporting animal research, and Sex and Gender in Research

Laboratory animals

The study was conducted in accordance with the approved animal use protocols (no. 18-03004 and 00001726) by the Institutional Animal Care and Use Committee (IACUC) at the University of Utah and the National Institute of Health Guide for the Care and Use of Laboratory Animals. All mice were kept in a pathogen-free animal facility and provided with a standard rodent chow diet. The housing facility maintained a controlled temperature (20-25 °C), a 12-hour light/dark cycle and a relative humidity of 30-70% to support the mice's circadian rhythm. The mice used in this study were derived from the C57BL/6J (B6) background obtained from Jackson Laboratory (RRID:IMSR_JAX:000664). Zbtb16 (luxoid) mice were also procured from Jackson Laboratory (RRID:IMSR_JAX:000100). Ddx4/Vasa-Cre (B6, RRID:IMSR_JAX:018980) was purchased from Jackson Laboratory. Sox3 floxed embryos (B6) were generously provided by Dr. Jeffrey Weiss. Sox3 conditional knockout (cKO) mice (B6), specifically targeting male germ cells, were generated using Ddx4-Cre mice. Male mice were randomly selected at the age of postnatal 7-90, and all the experiments used littermate controls or mice of the same ages.

Wild animals

No wild animals were used in this study.

Reporting on sex | All biological replicates employed in this study for male germ cell studies were derived from male C57BL/6J mice.

Field-collected samples | No field-collected samples were used in the study.

Ethics oversight | The authors confirm that all animal experiments were conducted in accordance with the approved animal use protocols (no. 18-03004 and 00001726) by the Institutional Animal Care and Use Committee (IACUC) at the University of Utah and the National Institute of Health Guide for the Care and Use of Laboratory Animals.

Note that full information on the approval of the study protocol must also be provided in the manuscript.

Plants

Seed stocks | N/A

Novel plant genotypes | N/A

Authentication | N/A

ChIP-seq

Data deposition

☒ Confirm that both raw and final processed data have been deposited in a public database such as GEO.

☒ Confirm that you have deposited or provided access to graph files (e.g. BED files) for the called peaks.

Data access links
May remain private before publication.

The datasets generated and/or analyzed during this study are publicly available on the GEO database under the following accession numbers: bulk RNA-seq and ChIP-seq data (GSE202819) and Hi-C data (GSE244681). All data sources, including the mm10 genome assembly, are appropriately cited and referenced.

Files in database submission

ChIPSeq_P7_Testis_ZBTB16_rabbit,
ChIPSeq_P7_Testis_ZBTB16_goat,
ChIPSeq_P7_Testis_SOX3,
ChIPSeq_P7_Testis_SALL4,
ChIPSeq_P7_Testis_ZBTB16_ChIPNexus,
ChIPSeq_P7_Testis_SOX3_ChIPNexus,
ChIPSeq_P7_Testis_SALL4_ChIPNexus,
ChIPSeq_P7_THY1+_uSPG_H3K4me3,
ChIPSeq_P7_THY1+_uSPG_H3K9me3,
ChIPSeq_P7_THY1+_uSPG_H3K27me3,
ChIPSeq_P7_Testis_WT_SALL4,
ChIPSeq_P7_Testis_WT_SOX3,
ChIPSeq_P7_Testis_WT_Pan-RNAPol2,
ChIPSeq_P7_Testis_WT_RNAPol2Ser5P,
ChIPSeq_P7_Testis_WT_RNAPol2Ser2P,
ChIPSeq_P7_Testis_Null_SALL4 and
ChIPSeq_P7_Testis_Null_SOX3

Genome browser session
(e.g. UCSC)

https://genome.ucsc.edu/s/jaruvy/ZBTB16_SOX3_SALL4

Methodology

Replicates | The ChIP-seq and ChIP-nexsus experiments for ZBTB16, SALL4, and SOX3 were conducted with two independent biological replicates.

Sequencing depth

Title Total reads Unique reads Sequence Length single or paired-end
ChIPSeq_P7_Testis_Input_rep1 37,594,101 25,526,801 50 single-end
ChIPSeq_P7_Testis_ZBTB16_rabbit_rep1 36,050,671 19,284,944 50 single-end
ChIPSeq_P7_Testis_ZBTB16_goat_rep1 28,832,621 18,973,252 50 single-end
ChIPSeq_P7_Testis_SOX3_rep1 32,153,478 16,132,382 50 single-end
ChIPSeq_P7_Testis_SALL4_rep1 39,298,208 20,173,212 50 single-end
ChIPSeq_P7_Testis_ZBTB16_ChIPNexus_rep1 30,444,948 78,929 50 single-end
ChIPSeq_P7_Testis_SOX3_ChIPNexus_rep1 27,835,503 37,308 50 single-end
ChIPSeq_P7_Testis_SALL4_ChIPNexus_rep1 27,824,153 87,230 50 single-end
ChIPSeq_P7_Testis_Input_rep2 41,302,839 27,994,685 50 single-end

ChIPSeq_P7_Testis_ZBTB16_rabbit_rep2 34,616,014 18,543,562 50 single-end
ChIPSeq_P7_Testis_ZBTB16_goat_rep2 34,186,714 20,233,831 50 single-end
ChIPSeq_P7_Testis_SOX3_rep2 38,718,666 21,940,574 50 single-end
ChIPSeq_P7_Testis_SALL4_rep2 36,166,684 20,280,165 50 single-end
ChIPSeq_P7_Testis_ZBTB16_ChIPNexus_rep2 27,091,147 54,720 50 single-end
ChIPSeq_P7_Testis_SOX3_ChIPNexus_rep2 27,867,097 23,303 50 single-end
ChIPSeq_P7_Testis_SALL4_ChIPNexus_rep2 27,867,628 380,585 50 single-end
ChIPSeq_P7_THY1+_uSPG_Input_rep1 36,682,065 26,816,768 50 single-end
ChIPSeq_P7_THY1+_uSPG_H3K4me3_rep1 35,641,326 19,701,827 50 single-end
ChIPSeq_P7_THY1+_uSPG_H3K9me3_rep1 36,129,820 12,844,452 50 single-end
ChIPSeq_P7_THY1+_uSPG_H3K27me3_rep1 36,620,517 26,547,448 50 single-end
ChIPSeq_P7_Testis_WT_SALL4_rep1 39,614,022 33,732,649 150 paired-end
ChIPSeq_P7_Testis_WT_SOX3_rep1 29,288,746 12,735,078 150 paired-end
ChIPSeq_P7_Testis_WT_TBP_rep1 32,049,918 25,715,535 150 paired-end
ChIPSeq_P7_Testis_WT_Pan-RNAPol2_rep1 30,628,382 27,873,006 150 paired-end
ChIPSeq_P7_Testis_WT_RNAPol2Ser5P_rep1 29,699,194 25,660,891 150 paired-end
ChIPSeq_P7_Testis_WT_RNAPol2Ser2P_rep1 37,513,828 33,793,427 150 paired-end
ChIPSeq_P7_Testis_WT_SALL4_rep2 37,565,462 32,063,498 150 paired-end
ChIPSeq_P7_Testis_WT_SOX3_rep2 39,382,316 33,015,219 150 paired-end
ChIPSeq_P7_Testis_WT_TBP_rep2 30,208,066 24,086,142 150 paired-end
ChIPSeq_P7_Testis_WT_Pan-RNAPol2_rep2 31,942,220 29,224,982 150 paired-end
ChIPSeq_P7_Testis_WT_RNAPol2Ser5P_rep2 35,961,962 32,402,681 150 paired-end
ChIPSeq_P7_Testis_WT_RNAPol2Ser2P_rep2 34,290,226 31,209,312 150 paired-end
ChIPSeq_P7_Testis_Null_SALL4_rep1 34,628,924 28,034,309 150 paired-end
ChIPSeq_P7_Testis_Null_SOX3_rep1 40,160,082 32,331,132 150 paired-end
ChIPSeq_P7_Testis_Null_SALL4_rep2 33,824,290 27,715,009 150 paired-end
ChIPSeq_P7_Testis_Null_SOX3_rep2 37,332,202 31,367,842 150 paired-end
ChIPSeq_P7_Testis_WT_Input_rep1 24,357,664 17,036,717 150 paired-end
ChIPSeq_P7_Testis_WT_Input_rep2 24,914,202 17,185,627 150 paired-end
ChIPSeq_P7_Testis_Null_Input_rep1 23,151,738 16,418,484 150 paired-end
ChIPSeq_P7_Testis_Null_Input_rep2 25,414,338 16,155,747 150 paired-end

Antibodies

Host organism Target Manufacturer Catalog number Application
rabbit H3K27me3 EMD Millipore Cat# 07-449 5 µl
rabbit H3K4me3 Active Motif Cat# 39159 3 µl
rabbit H3K9me3 Active Motif Cat# 39161 5 µl
mouse RNAPol2 Active Motif Cat# 39097 0.2 µg (1 µl)
rabbit RNAPol2 CTD phospho Ser5 Active Motif Cat# 39233 ChIP 5 µg (5 µl)
rabbit RNA Pol2 CTD phospho Ser2 Active Motif Cat# 61083 ChIP 5 µg (5 µl)
rabbit SALL4 Abcam Cat# ab29112 5 µg
mouse SOX3 Santa Cruz Cat# sc-101155 2.5 µg (25 µl)
rabbit SOX3 EMD Millipore Cat# AB5772 ChIP 2.5 µg (5 µl)
mouse TBP EMD Millipore Cat# MAB3658 AB_2200056 ChIP 1 µl
goat ZBTB16 Santa Cruz Cat# sc-11146 5 µg (25 µl)
rabbit ZBTB16 Santa Cruz Cat# sc-22839 5 µg (25 µl)

Peak calling parameters

Peak calling was performed using USeq8.9.6 packages and MACS (2.1.1) with the default parameters.

Data quality

Peaks with -10log10(q-Value) (QValFDR) greater than 30 (q<0.001) for USeq and 20 (q<0.01) for MACS were considered for downstream analyses.

Software

For ChIP-seq data analysis, the reads were aligned to the mouse reference genome (mm10) using Novoalign (http://novocraf.com) with the following parameters: -o SAM -r Random -H -a AGATCGGAAGAGCACACGTCTGAACTCCAGTCA, which includes adapter sequence removal. To ensure fair comparison across all datasets, PCR duplicates and all unmapped reads were removed using Picard MarkDuplicates (version 2.7.1; https://broadinstitute.github.io/picard/) and Samtools. Peak calling was performed using USeq8.9.6 packages and MACS (2.1.1) with the default parameters. Peaks with -10log10(q-Value) (QValFDR) greater than 30 (q<0.001) for USeq and 20 (q<0.01) for MACS were considered for downstream analyses. Replicate handing was modified based on a previous approach. Briefly, only peaks from the merged files of two/three replicates overlapped with at least 50% of peaks from the union of biological replicates. This was achieved using BEDTools intersect with parameters: -u -f 0.5. Moreover, genomic regions blacklisted in mice were removed from the peaks using BEDTools intersect with the parameter: -v. To annotate the peaks, ChIPseeker was used with the RefSeq gene list for genome version mm10. For ZBTB16, SALL4 and SOX3, promoters were defined as ±2 Kb from the transcription start site (TSS), and for histone modification, promoters were defined as ±1 Kb from the TSS. Additionally, annotated genes that were excluded from the merged replicates but overlapped with at least two biological replicates were included, and bidirectional promoter genes were manually added using BEDTools. For genome browser visualization, ChIP signals were first normalized to their corresponding inputs using USeq after peak calling. The snapshots for genome browser were captured using the IGV browser. To facilitate the analysis, we employed the deepTools suite, using the bamCompare module to ChIP signals against their input. The computeMatrix and plotHeatmap modules were used for ChIP-seq heatmaps and clustering, employing a reference-point approach. The spearman correlation between ChIP-seq samples was computed using the plotCorrelation module.

Flow Cytometry

Plots

Confirm that:

☒ The axis labels state the marker and fluorochrome used (e.g. CD4-FITC).

☒ The axis scales are clearly visible. Include numbers along axes only for bottom left plot of group (a 'group' is an analysis of identical markers).

☒ All plots are contour plots with outliers or pseudocolor plots.

☒ A numerical value for number of cells or percentage (with statistics) is provided.

Methodology

Sample preparation	To perform flow cytometry analysis, single cells were isolated from testes at P7 and resuspended in MACS separation buffer (Miltenyi Biotec, 130-091-221). The collected cells were subsequently stained with anti-THY1/CD90.2-PE-Cy7 (Thermo Fisher Scientific, 25-0902-81) and anti-KIT/CD117-PE (Thermo Fisher Scientific, 12-1171-81) antibodies, according to the manufacturer's instructions, for a duration of 15 minutes at 4 °C. Following this, the cells were fixed in a solution of 4% formaldehyde/PBS for 20 minutes on ice. After appropriate washes, the cells were subjected to staining with DAPI solution (0.1% Triton X-100 and 10 µg/ml of DAPI in PBS) and incubated overnight at 4 °C.
Instrument	FACS Canto Scan (BD Biosciences)
Software	DATA collection: FACSDiva version 8.01 (BD Biosciences)<br>Data analysis: FlowJo (v9.9, BD Biosciences)
Cell population abundance	Populations were sorted as depicted in Extended Data Fig.8c. The FACS Canto Scan is not suitable for analyzing cell population abundance from post-sort fractions. Sort purity was not validated through reanalysis, but antibody specificity was confirmed using unstained cells and single-stained cells. Sort efficiency was typically 67% (actual number of cells vs. sorter counts). The markers used for sorting were differentially expressed by RNA, as illustrated in Extended Data Fig. 1c, indicating that the sorted populations were pure.
Gating strategy	Initially, potential cells were identified using forward scatter (FSC) area and back scatter (BSC) area. Subsequently, potential doublets were eliminated based on BSC and FSC signal width. Finally, undifferentiated spermatogonia and differentiating spermatogonia were separated using THY1 and KIT antibodies, followed by the analysis of DNA contents for cell cycle assessment.

☒ Tick this box to confirm that a figure exemplifying the gating strategy is provided in the Supplementary Information.
