# Supplementary figures and images for: ZBTB16/PLZF regulates juvenile spermatogonial stem cell development through an extensive transcription factor poising network
Source: Nat Struct Mol Biol. 2025 Mar 3;32(7):1213–26. doi: 10.1038/s41594-025-01509-5 (PMC12263333; doi:10.1038/s41594-025-01509-5)

Extended Data Fig.1c

a. Rabbit anti-ZBTB16

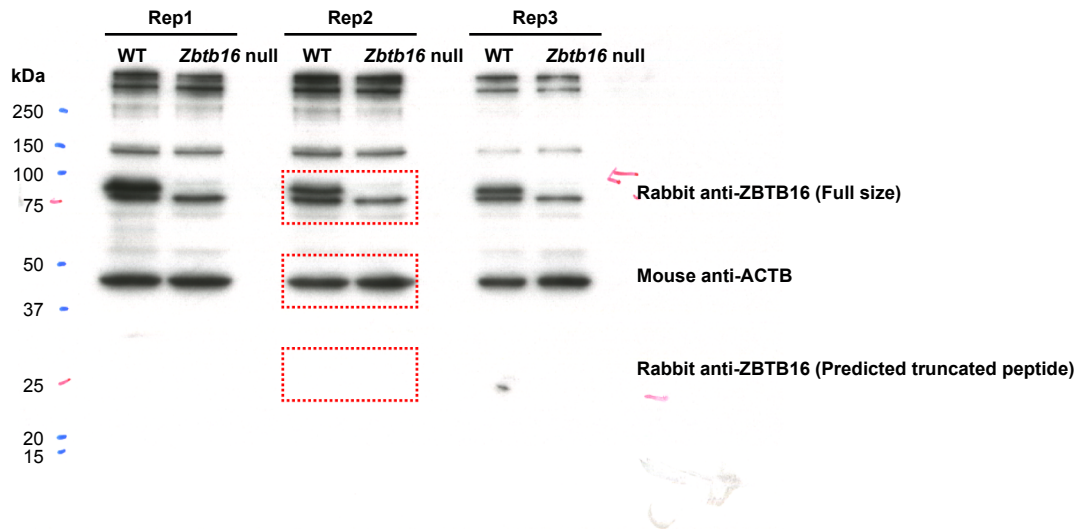

b. Goat anti-ZBTB16

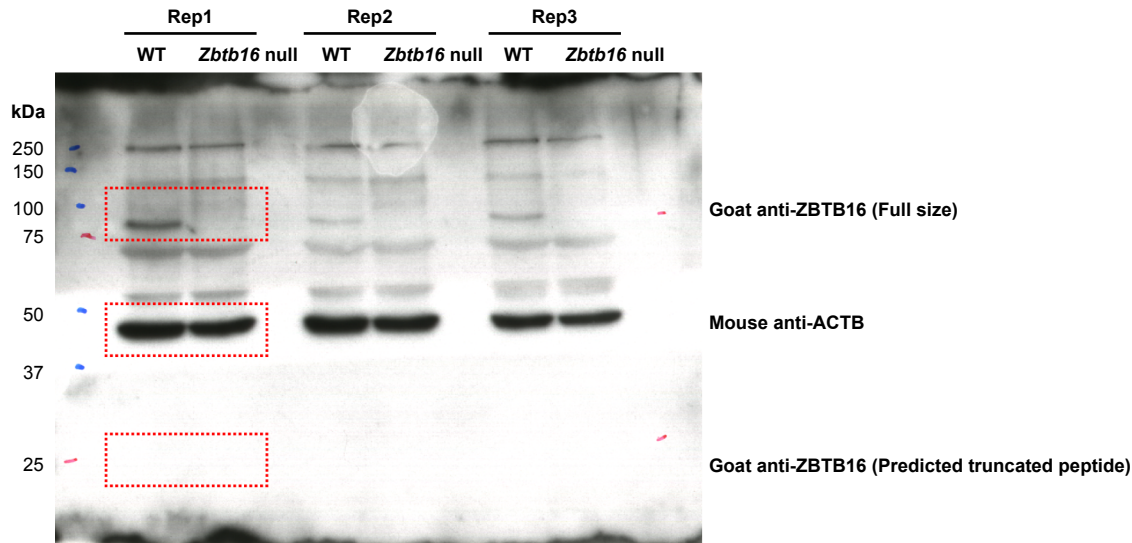

Supplement: Supplementary file 10 — Unprocessed western blots. [file 41594_2025_1509_MOESM10_ESM.pdf]
